# A novel hydrogen peroxide evolved CHO host can improve the expression of difficult to express bispecific antibodies

**DOI:** 10.1002/bit.27744

**Published:** 2021-03-25

**Authors:** Rajesh K. Mistry, Emma Kelsall, Si Nga Sou, Harriet Barker, Mike Jenns, Katie Willis, Fabio Zurlo, Diane Hatton, Suzanne J. Gibson

**Affiliations:** ^1^ Cell Culture and Fermentation Sciences, BioPharmaceuticals Development, R&D, AstraZeneca Cambridge UK; ^2^ Kymab Ltd, Cell Line Development, Biopharmaceutical Development, Kymab, Babraham Research Campus Cambridge UK; ^3^ Department of Life Sciences Imperial College London Berkshire UK

**Keywords:** bispecific antibody, evolved host, hydrogen peroxide, redox

## Abstract

The manufacture of bispecific antibodies by Chinese hamster ovary (CHO) cells is often hindered by lower product yields compared to monoclonal antibodies. Recently, reactive oxygen species have been shown to negatively impact antibody production. By contrast, strategies to boost cellular antioxidant capacity appear to be beneficial for recombinant protein expression. With this in mind, we generated a novel hydrogen peroxide evolved host using directed host cell evolution. Here we demonstrate that this host has heritable resistance to hydrogen peroxide over many generations, displays enhanced antioxidant capacity through the upregulation of several, diverse antioxidant defense genes such as those involved in glutathione synthesis and turnover, and has improved glutathione content. Additionally, we show that this host has significantly improved transfection recovery times, improved growth and viability properties in a fed‐batch production process, and elevated expression of two industrially relevant difficult to express bispecific antibodies compared to unevolved CHO control host cells. These findings demonstrate that host cell evolution represents a powerful methodology for improving specific host cell characteristics that can positively impact the expression of difficult to express biotherapeutics.

## Graphical abstract

A novel H_2_O_2_ evolved CHO host was generated and characterised in this study. This host displays enhanced antioxidant capacity through the upregulation of several, diverse antioxidant defence genes such as those involved in glutathione synthesis and turnover and has improved glutathione content. Additionally, this host has significantly improved transfection recovery times, improved growth and viability properties in a fed batch production process and elevated expression of two industrially relevant difficult to express bispecific antibodies compared to unevolved CHO control host cells.

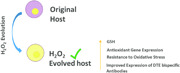

## 1 INTRODUCTION

Recently, an interest in the cellular redox state and its effects on recombinant protein production has emerged (Handlogten et al., 2017, 2018, 2020; Orellana et al., 2015). Reactive oxygen species (ROS) are partial reduction products of molecular oxygen generated as a result of mitochondrial oxidative phosphorylation and oxidative protein folding within the endoplasmic reticulum (ER; Chevallier et al., 2020; Santos et al., 2009; Turrens, 2003; Zeeshan et al., 2016). Chinese hamster ovary (CHO) cells represent the mammalian cell type of choice for the expression of monoclonal antibodies (mAb) as well as a growing repertoire of diverse, nonnative biopharmaceutical molecules such as bispecific antibodies (BisAbs; Kim et al., 2012; Wang et al., 2019). Recombinant CHO cells are postulated to produce high levels of ROS during the bioreactor process which can lead to oxidative stress and therefore suboptimal cell culture performance and lower antibody titers (Handlogten et al., 2018).

To overcome the deleterious effects of exacerbated ROS production and ensure the maintenance of redox homeostasis, mammalian cells have evolved a complex antioxidant defense system. These antioxidants fall into two broad categories; enzymatic and nonenzymatic. Enzymatic antioxidants include catalase, superoxide dismutase, peroxiredoxins, thioredoxins, glutathione reductases, and glutathione peroxidases. Nonenzymatic antioxidants include several small molecules such as vitamin E but are largely centered around glutathione (GSH; Valko et al., 2007). GSH is a tripeptide that is present in all cell types at millimolar concentrations and acts as a major redox buffer affecting a broad range of intracellular systems (Forman et al., 2009). Antioxidants are thought to be important for recombinant protein expression. Indeed, the depletion of the reduced form of GSH (active GSH) has been linked to decreased specific productivity (qP) of manufacturing cell lines (Handlogten et al., 2020) and proteomic work demonstrated that high antibody‐producing CHO cell lines upregulated GSH biosynthetic pathways (Orellana et al., 2015). These data are supported by observations that high producer cell lines had increased cellular GSH content (Chong et al., 2012). Consistently, the modulation of GSH synthetic enzymes, through targeted genetic overexpression, was shown to improve mAb titers (Orellana et al., 2017). In addition to GSH, other antioxidants such as thioredoxin reductase 1 and peroxiredoxin 6 were shown to be elevated by depletion of microRNA 23 and linked to improved recombinant protein expression (Kelly et al., 2015). More recently, the transcription factor Forkhead BoxA1 (Foxa1) has been linked to improved expression of difficult to express (DTE) antibodies through a mechanism involving reduced oxidative stress (Berger et al., 2020). Taken together, the upregulation of antioxidants appears to be beneficial for the expression of recombinant proteins in CHO cells.

The development of novel DTE antibodies is often hindered by manufacturing challenges resulting from low product yields (Spiess et al., 2015) which have been associated with high levels of cellular stress, including oxidative stress (Chevallier et al., 2020). Rational genetic engineering approaches involving the manipulation of specific genes through overexpression or targeted genetic ablation to alter subcellular processes have been implemented to relieve production bottlenecks and produce more predictable and robust cell lines. To date, this strategy has been employed to alter diverse subcellular processes including cell cycle (Fussenegger et al., 1998), metabolism (Fogolin et al., 2004), protein secretion (Mohan et al., 2007), and importantly, cellular redox (Banmeyer et al., 2004; Orellana et al., 2017; Warner et al., 1993). Although these strategies have been used to boost cell line characteristics to moderate success, the dawn of the “omics” era suggests that targeting specific genes in this way may not be as effective as originally hypothesized. This is due, in part, to the complex interplay of intracellular pathways that give rise to dynamic web‐like interaction systems that are capable of compensating for the misexpression of an individual gene. In fact, using a method that induces global cellular changes as opposed to those that target individual genes may prove more effective for boosting productivity. An example of this is directed host cell evolution, a technique that can offer a relatively unbiased, simple but effective method of engineering CHO cells such that they are evolved to be endowed with specific characteristics that make them superior to their predecessor. With this in mind, we evolved our suspension adapted CHO host cells in the presence of hydrogen peroxide (H_2_O_2_) to select for cells conferring resistance to oxidative stress. Here, we describe the generation and characterization of a novel H_2_O_2_ evolved CHO host and the evaluation of these cells for the expression of two industrially relevant, DTE BisAbs compared to our non‐evolved CHO host.

## 2 MATERIALS AND METHODS

### 2.1 CHO host cell evolution using hydrogen peroxide

Suspension adapted CHO‐K1 cells (CHO Control; AstraZeneca) were revived into CD CHO medium (Life Technologies) supplemented with 6 mM l‐glutamine (Life technologies) and passaged three times at 0.2 × 10^6^  cells/ml in a 30 ml culture volume. Once at 99% viability, the cells were incubated for 1 h with 14 mM H_2_O_2_ (Sigma‐Aldrich) before centrifugation at 130*g* for 5 min and resuspension in 30 ml fresh CD CHO supplemented with 6 mM l‐glutamine. CHO cells were left to recover until cells reached 70% viability. This process was repeated an additional three times resulting in a total of four exposures to 14 mM H_2_O_2_, in which cells gradually recovered over a period of 10–20 days, with the medium being replenished periodically until cells reached 70% viability. The cells were then subjected to one round of 18.5 mM H_2_O_2_ treatment following the method above until cells reached 90% viability. Finally, the cells were incubated with 37 mM H_2_O_2_ for 1 h before centrifugation at 130*g* for 5 min and resuspension in 30 ml fresh CD CHO supplemented with 6 mM l‐glutamine and left to recover until >90% viable on Day 24. During this recovery period, the culture medium was replenished on Day 12 and the cells were diluted to 0.3 × 10^6^ cells/ml on Day 21 with fresh medium to aid recovery. Once at >90%, the H_2_O_2_ treated CHO cells were cryopreserved. All experiments were performed using this cryopreserved cell stock.

### 2.2 Cell lines and culture conditions

CHO Control or H_2_O_2_ evolved host cells (AstraZeneca) were maintained in either CD CHO medium (Life Technologies) or AstraZeneca proprietary medium, both supplemented with 6 mM l‐glutamine (Life Technologies). Stably transfected CHO cells were grown in AstraZeneca proprietary medium supplemented with methionine sulfoximine (MSX; Sigma‐Aldrich). Cell cultures were grown in polycarbonate Erlenmeyer flasks with vented caps (Corning) in a humidified incubator at 36.5°C, 6% CO_2_ with agitation at 140 rpm, 25 mm rotation diameter as required.

### 2.3 BisAb expression plasmids

The BisAb A and BisAb B stable expression plasmids were modified from transient expression plasmids (Daramola et al., 2014; Persic et al., 1997) and encoded both the BisAb Heavy Chain (Hc) and Light Chain (Lc) in addition to the glutamine synthetase (GS) selectable marker and were constructed by standard restriction enzyme digestion and ligation methods.

### 2.4 Production of BisAbs A and B by stable CHO pools

Stable CHO pools expressing BisAbs were generated by transfecting either CHO Control or H_2_O_2_ evolved host cells with a plasmid encoding either BisAb A or B and the GS selectable marker using an Amaxa nucleofector and reagents (Lonza). The transfected cells were selected and maintained in CD CHO in the presence of 50 µM MSX. Pools were counted regularly during the course of transfection recovery using a Vi‐Cell XR Cell Viability Analyzer (Beckman Coulter). Pools of cells were expanded and used for the production of BisAb A and BisAb B in a 12‐day fed‐batch process using AstraZeneca proprietary medium. The medium was supplemented with bolus additions of an AstraZeneca proprietary nutrient feed added over the course of the culture period. Glucose and lactate were monitored throughout the fed‐batch process using a YSI (2900D, YSI Inc). Cell culture medium was clarified by centrifugation and then BisAbs were quantified by protein‐A HPLC affinity chromatography on an Agilent 1260 Infinity series (Agilent Technologies) by comparing the peak size from each sample with a calibration curve. Qp was calculated as follows: QP = Th/CCTf (where Th is the Harvest Titer and CCTf is the calculated Cumulative Cell Time on the last day of the culture). CCTi = ((di − di‐1) × (VCNi + VCNi‐1)/2) + CCTi‐1 (where *d* is the day of the culture, VCN is the viable cell count and i is the day of VCN sampling during the course of the culture).

### 2.5 Glutathione assays

Relative changes in intracellular total glutathione (total GSH) and oxidized glutathione (GSSG) were determined with GSH/GSSG‐Glo™ Assay Kit (Promega) according to the manufacturer's instructions. Briefly, CHO Control or H_2_O_2_ evolved host cells in culture were harvested and resuspended in fresh AstraZeneca proprietary medium supplemented with 6 mM l‐glutamine (untransfected hosts) or 50 µM MSX (transfected pools). Cells were seeded at 10,000 cells/well in a white 96‐well luminometer‐compatible plate (medium‐only wells were used for background luminescence detection). A 25 µl volume of either total glutathione lysis reagent or oxidized glutathione lysis reagent was added to cell‐containing wells and incubated at room temperature on a plate shaker for 5 min. Then, 50 µl of freshly prepared luciferin generation reagent was added to all wells followed by a 30 min incubation at room temperature. Finally, 100 µl of luciferin detection reagent was added to each well and incubated for 15 min. Luminescence was measured using an EnVision Microplate Luminometer (PerkinElmer). The analysis was performed according to the manufacturer's instructions.

### 2.6 Chemstress assays

Chem stress assays were performed according to the manufacturer's instructions (ChemStress®, Valitacell Ltd). In brief, CHO or H_2_O_2_ evolved host cells were seeded into Valitacell ChemStress plates at 18,000 cells/well in 90 µl AstraZeneca proprietary medium supplemented with 6 mM l‐glutamine. A control well was incubated with medium alone. Plates were incubated for 72 h in a static incubator at 36.5°C, 6% CO_2_. Following this, 10 µl of neat PrestoBlue dye (Thermo Fisher Scientific) was added to all wells before plates were mixed for 20 s and incubated for a further 30 min at 36.5°C, 6% CO_2_. Plates were analyzed using a PHERAstar plate reader (BMG LABTECH with preconfigured protocols (excitation 560 nm, emission 590 nm). Data were analyzed using the ValitaAPP software (Valitacell Ltd).

### 2.7 MSB survival assays

CHO Control or H_2_O_2_ evolved host cells expressing BisAb A or B were seeded at 0.3 × 10^6^ cells/ml into 30 ml of AstraZeneca proprietary medium supplemented with 50 µM MSX. A total of 6 µM menadione sodium bisulfite (MSB; Sigma‐Aldrich) or water was added to test and control cultures, respectively. Cells were assessed for viability and viable cell number (VCN) using a Vi‐Cell XR Cell Viability Analyzer at 24, 48, and 72 h post addition of MSB or water.

### 2.8 RNA analyses

RNA was extracted from CHO control or H_2_O_2_ evolved host cells (both untransfected and transfected) using the Qiagen RNA Isolation Kit according to the manufacturer's instructions. cDNA was generated by reverse transcribing 3 µg of RNA using the SuperScript™ IV First‐Strand Synthesis System (Thermo Fisher Scientific) according to the manufacturer's instructions. qPCR reactions were made up in a final volume of 20 µl using 3 ng cDNA and 1 µl of each 20× TaqMan Assay probe (qPCR probes are detailed in Table 1; Thermo Fisher Scientific). qPCR was performed on the QuantStudio 12K Flex Real‐Time PCR System (Applied Biosciences). Relative gene expression was calculated using the $2^{‐\Delta \Delta C_{t}}$ method, the MMADHC reference gene was selected from Brown et al. (2018).

**Table 1:** Primer probes used for qPCR analysis of gene expression

Primer name	Catalog number	Reference number
Catalase	4351372	Cg04624486_m1
GCLM	4351372	Cg04497880_m1
GPrx1	4351372	Cg04422105_g1
GSS	4351372	Cg04491342_m1
xCT (slc7a11)	4351372	Cg04496729_m1
MMADHC	4351372	Cg04467875_m1

## 3 RESULTS

### 3.1 Directed‐evolution of CHO cells with H_2_O_2_ results in a novel, oxidative‐stress resistant host

**Figure 1:**
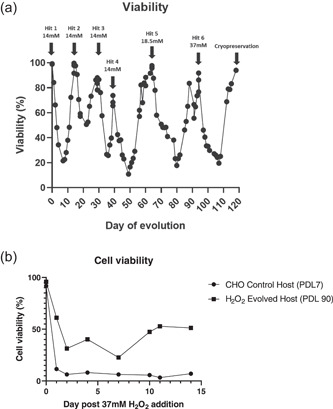
Creation of an H_2_O_2_ evolved host. A viability plot tracking cell recovery during the H_2_O_2_ host evolution process (a) (arrows indicate the day and concentration of H_2_O_2_ addition, black circles represent cell viability counts). The H_2_O_2_ evolved host were passaged to 29 and 90 PDL, and CHO control host to 7 PDL respectively in CD‐CHO supplemented with 6 mM glutamine after which all hosts were re‐challenged with 37 mM H_2_O_2_ and viabilities recorded (viability measured on Days 1, 2, 4, 7, 10, 11, and 12) (b). *N* = 1. CHO, Chinese hamster ovary; PDL, population doubling level

To generate a novel CHO host with improved resistance to oxidative stress, AstraZeneca proprietary CHO control host cells were evolved through multiple rounds of successive H_2_O_2_ exposure followed by recovery until host cells demonstrated survival in the presence of 37 mM H_2_O_2_ (Figure 1a). To assess that the evolutionary changes induced by H_2_O_2_ treatment were maintained, these cells were passaged to 90 population doubling levels and subjected to rechallenge with 37 mM H_2_O_2_. The H_2_O_2_ evolved host cells demonstrated improved survival (~50% viability) compared to CHO control cells that had also undergone H_2_O_2_ treatment (Figure 1b) indicating long‐term heritable resistance to high concentrations of H_2_O_2_.

### 3.2 H_2_O_2_ evolved CHO cells have improved survival when grown in the presence of redox chemical stressors

**Figure 2:**
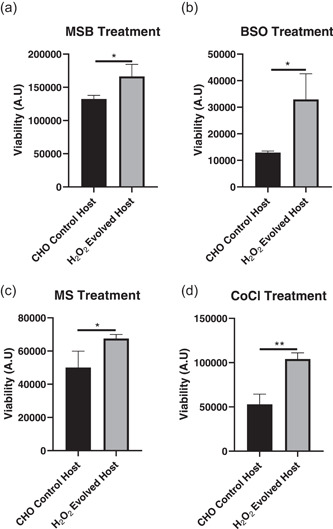
CHO host cell survival in response to redox stressors using Chemstress plates. A comparison of relative host cell viabilities following 72‐h incubation with menadione sodium bisulfite (MSB) (a), buthionine sulfoximine (BSO) (b), mercaptosuccinic acid (MS) (c), and cobalt chloride (CoCl) (d) between CHO control and H_2_O_2_ evolved host cells. The graphs show the mean ± *SD, N* = 3 in all cases, statistics determined using an unpaired *t*‐test. CHO, Chinese hamster ovary. *p < 0.05, **p < 0.005

To further confirm the H_2_O_2_ evolved host's ability to resist oxidative stress, these cells were evaluated for survival in response to various chemical compounds that mimicked the redox stress encountered by cells in the bioreactor process (Beck et al., 2011; Dunning et al., 2013; Lee et al., 1992; Zou et al., 2001). Here, H_2_O_2_ evolved host cells demonstrated significantly improved viabilities following 72 h growth in the presence of MSB, buthionine sulfoximine (BSO), mercaptosuccinic acid (MS), and cobalt chloride (CoCl) compared to CHO control cells (Figure 2a–d) demonstrating that H_2_O_2_ evolved host cells had developed resistance to diverse redox stressors.

### 3.3 H_2_O_2_ evolved CHO cells have improved antioxidant capacity

**Figure 3:**
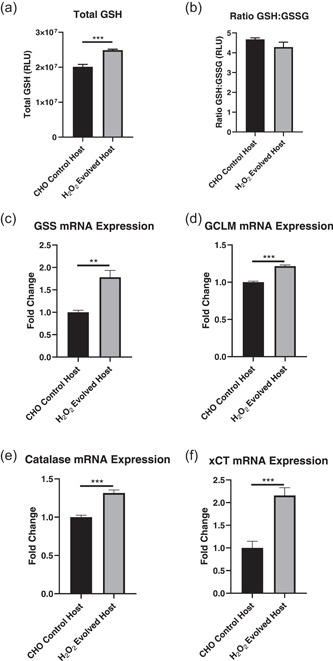
Characterization of the antioxidant capacity of H_2_O_2_ evolved host cells. A comparison of total GSH (a) and the ratio of GSH:GSSG (b) between CHO control and H_2_O_2_ evolved hosts. Relative mRNA expression of glutathione synthetase (GSS) (c), gamma‐glutamylcysteine ligase modulator subunit (GCLM) (d), catalase (e), and xCT in CHO control and H_2_O_2_ evolved host cells. All qPCR data were normalized to MMADHC mRNA expression and *SD* calculated on fold change relative to control. The graphs show the mean ± *SD, N* = 3 in all cases, statistics determined using an unpaired *t*‐test. CHO, Chinese hamster ovary. **p < 0.005, ***p < 0.0005

The ability of the H_2_O_2_ evolved host to survive in the presence of several redox stressors suggests that H_2_O_2_‐induced evolution may have augmented antioxidant defense pathways within the cell. To better understand this, we sought to evaluate this host based on its GSH content and transcriptional changes in antioxidant defense genes. Here, early passage H_2_O_2_ evolved host cells were shown to have significantly elevated total GSH content with respect to CHO control cells, however, the ratio of total to oxidized GSH (GSH:GSSG) remained unchanged (Figure 3a,b). Next, the expression of a panel of antioxidant defense genes was compared between the H_2_O_2_ evolved host and CHO control cells. Here, the H_2_O_2_ evolved host demonstrated significantly elevated expression of genes involved in GSH synthesis (GSS, GCLM, Figure 3c,d), an observation consistent with the elevated total GSH content of the H_2_O_2_ evolved host. In addition, the H_2_O_2_ evolved host demonstrated significantly elevated expression of genes involved in H_2_O_2_ elimination (catalase, Figure 3e) and cellular cysteine import (xCT, Figure 3f), indicating further improvements of antioxidant capacity in this host.

### 3.4 H_2_O_2_ evolved host cells expressing DTE BisAbs maintain resistance to oxidative stress

**Figure 4:**
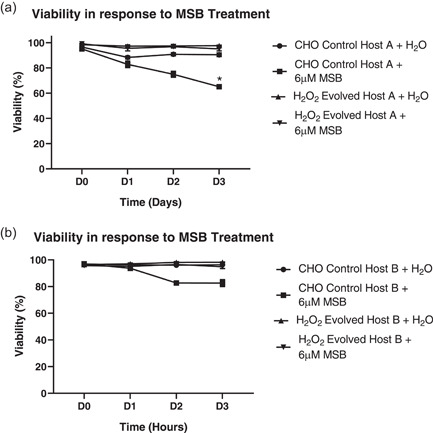
Survival of transfected hosts expressing BisAb A and B in response to menadione sodium bisulfite treatment (MSB). Viability plots of CHO control host A and H_2_O_2_ evolved host A (a) and CHO control host B and H_2_O_2_ evolved host B (b) in response to 6 µM MSB or H_2_O treatment for 72 h. The graphs show the mean + *SD, N* = 3 in all cases, statistics determined using a one‐way ANOVA and a Tukey's multiple comparison test. CHO, Chinese hamster ovary. *p < 0.05 (compares CHO control host A + 6 µM MSB and H_2_O_2_ evolved host A + 6 µM MSB)

To investigate whether the H_2_O_2_ evolved host, when expressing DTE BisAbs, maintained resistance to oxidative stress, H_2_O_2_ evolved and CHO control host cells were stably transfected with plasmid DNA encoding BisAbs A and B to yield stable pools (resulting transfected cell populations are denoted: H_2_O_2_ evolved host A or B and CHO control host A or B). Expressing pools were compared for survival in the presence of the widely used prooxidant, MSB. H_2_O_2_ evolved hosts A and B treated with MSB retained viabilities comparable with H_2_O treated controls. By contrast, CHO control hosts A and B treated with MSB displayed a reduction in viability at Day 3 posttreatment to 65% and 82%, respectively (Figure 4a,b).

### 3.5 H_2_O_2_ evolved DTE BisAb‐expressing cells maintain an elevated antioxidant capacity

**Figure 5:**
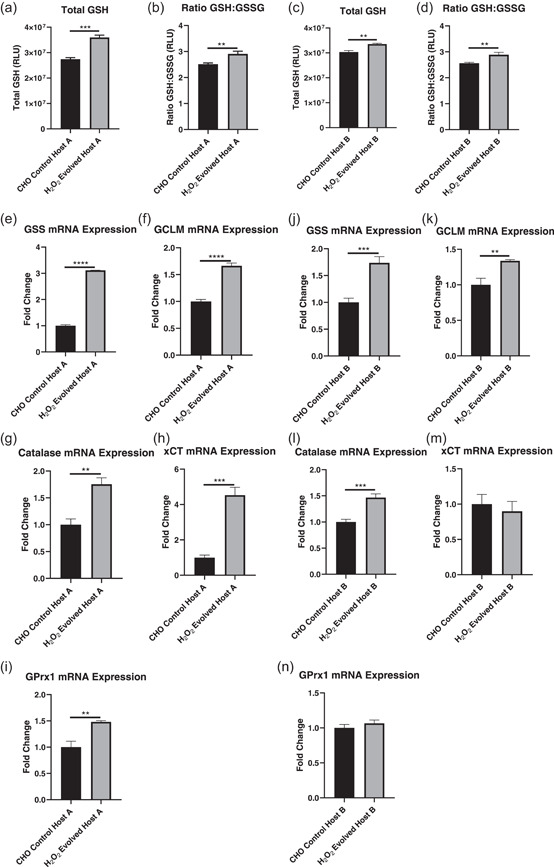
Characterization of the antioxidant capacity of transfected H_2_O_2_ evolved host cells. Left panel: Comparison of total GSH (a) and the ratio of GSH:GSSG (b). Relative mRNA expression of glutathione synthetase (GSS) (e), gamma‐glutamylcysteine ligase modulator subunit (GCLM) (f), xCT (g) catalase (h), and glutathione peroxidase1 (GPrx1) (i) in CHO control host A and H_2_O_2_ evolved host A. Right Panel: Comparison of total GSH (c) and the ratio of GSH:GSSG (d). Relative mRNA expression of GSS (j), GCLM (k), xCT (l) catalase (m), and GPrx1 (n) in CHO control host B and H_2_O_2_ evolved host B. All qPCR data were normalized to MMADHC mRNA expression and *SD* calculated on fold change relative to control. The graphs show the mean ± *SD, N* = 3 in all cases, statistics determined using an unpaired *t*‐test. CHO, Chinese hamster ovary. **p < 0.005, ***p < 0.0005, **** = *p* < 0.00005

To investigate whether the elevated antioxidant phenotypes observed in the untransfected H_2_O_2_ evolved host cells were maintained in H_2_O_2_ evolved hosts A and B, both GSH content and antioxidant defense gene expression were assessed. Here, both H_2_O_2_ evolved hosts A and B displayed a significant upregulation in total GSH content as well as improved ratios of GSH:GSSG, however, this increase was less apparent in H_2_O_2_ evolved host B (Figure 5a–d). Second, H_2_O_2_ evolved host pools were assessed for the expression of several antioxidant defense genes. H_2_O_2_ evolved host A demonstrated significant elevations in GSS, GCLM, catalase, and xCT as well as glutathione peroxidase 1 (GPrx1) compared to CHO control host A (Figure 5e–i). H_2_O_2_ evolved host B also showed significant elevations in GSS, GCLM, and catalase, however, xCT and GPrx1 remained unchanged with respect to CHO control Host B (Figure 5j–n). Taken together these data indicate that the improvements in antioxidant capacity observed in the untransfected H_2_O_2_ evolved host (Figure 3a–f) are maintained upon expression of both DTEs BisAbs and have the further benefit of improved GSH:GSSG ratios.

### 3.6 The H_2_O_2_ evolved host demonstrates improved platform performance compared to CHO control cells

#### 3.6.1 Improved recovery post‐transfection

**Figure 6:**
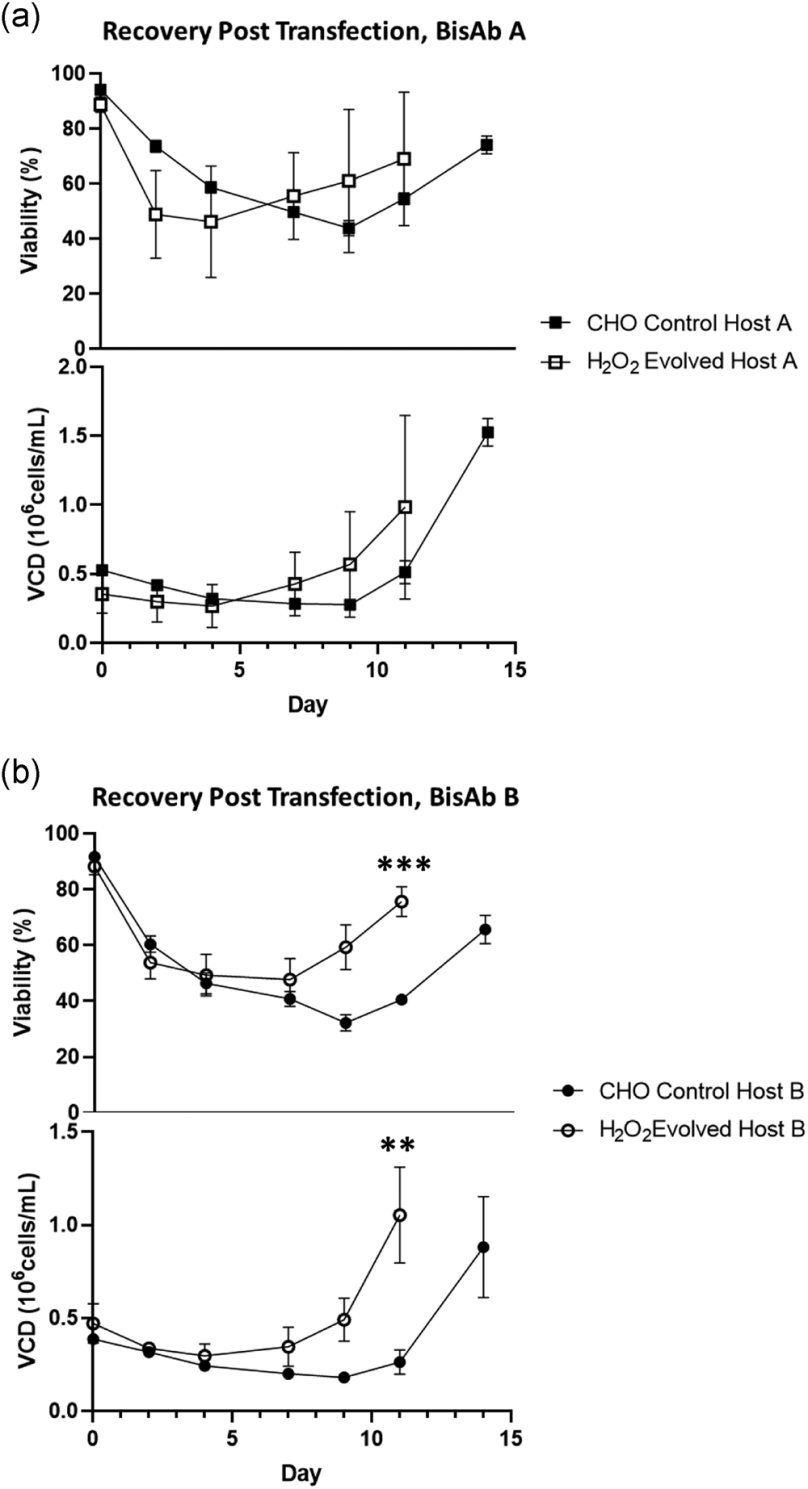
Recovery of stable pools post‐transfection. Cell viability and viable cell density (VCD) of H_2_O_2_ evolved host A and CHO control host A (a) and cell viability and VCD of H_2_O_2_ evolved host B and CHO control host B (b) were monitored after transfection. Three pools were generated for each molecule and each host. Error bars are mean viability or VCN ± *SD. N* = 3 in all cases, statistics determined for the Day 11‐time point using an unpaired *t*‐test. CHO, Chinese hamster ovary. *p < 0.05, ***p < 0.0005

Stable transfection of CHO cells using electroporation followed by MSX selection is stressful leading to cell death and cell recovery times of 14 days or more. To evaluate the H_2_O_2_ host performance during transfection recovery, cell viability and viable cell density (VCD) were monitored following transfection. Three expressing pools were generated per molecule for each host. For the H_2_O_2_ evolved host transfected with plasmid DNA encoding BisAb A, two of the pools reached viabilities of 82%–84% and VCDs of 1.2–1.50 × 10^6^ cells/ml on Day 11 post‐transfection and were transferred to shaking cultures. In contrast, CHO control hosts transfected with BisAb A reached a similar level of recovery (VCD: 1.4–1.6 × 10^6^ cells/ml with viabilities of 71%–78%, Figure 6a) at Day 14 post‐transfection. The same trend was seen with BisAb B where all three of the H_2_O_2_ evolved host transfected pools reached 71%–82% viabilities and VCDs of 0.88–1.35 × 10^6^ cells/ml on Day 11 post‐transfection, a significant improvement over the equivalent CHO control host transfected pools which reached 60%–70% viability and 0.65–1.18 × 10^6^ cells/ml at Day 14 before being transferred to shaking cultures (Figure 6b). These data further demonstrate that the H_2_O_2_ evolved host has increased resistance to cellular stress compared to the unevolved CHO control cells.

#### 3.6.2 BisAb‐expressing H_2_O_2_ evolved pools demonstrate improved performance in a fed‐batch process

**Figure 7:**
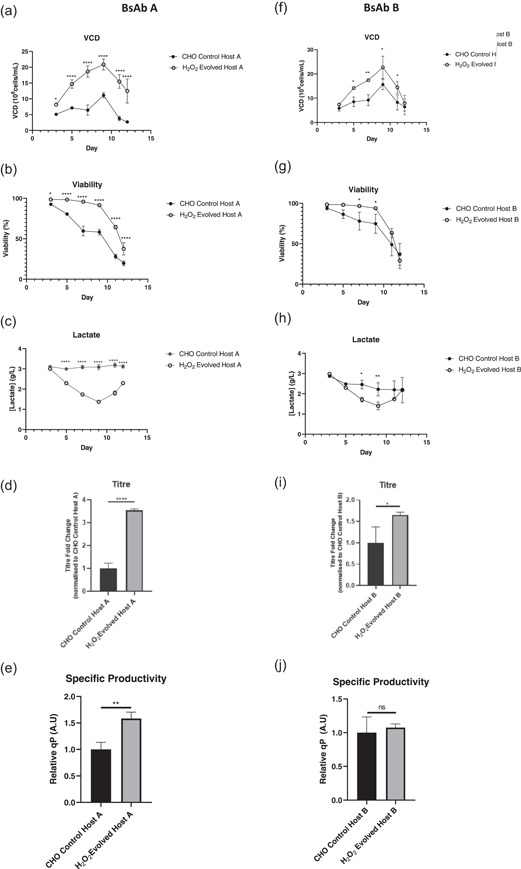
Stable pool performance during the fed‐batch process. Left panel: A comparison of viable cell density (VCD) (a), Viability (b), Lactate (c), Titer (d), and cell‐specific productivity (qP) (e) between CHO control A and H_2_O_2_ evolved host A. Right panel: A comparison of VCD (f) Viability (g), Lactate (h), Titer (I), and qP (j) between CHO control B and H_2_O_2_ evolved host B. A total of three pools expressing each molecule were evaluated for each host. Error bars are mean ± *SD. N* = 3 in all cases, statistics determined using an unpaired *t*‐test for titer and qP and multiple *t*‐tests on VCD, viability, and lactate time courses comparing each time point individually. CHO, Chinese hamster ovary; ns, not significant. *p < 0.05, **p < 0.005, ***p < 0.0005, ****p < 0.00005

To assess host cell performance during BisAb production, a 12‐day fed‐batch process was performed with H_2_O_2_ evolved hosts A and B alongside CHO control hosts A and B. Strikingly, growth rates of H_2_O_2_ evolved hosts A and B were dramatically improved compared to CHO control pools irrespective of the BisAb being expressed (Figure 7a,f). Peak VCN for H_2_O_2_ evolved host A was 20 × 10^6^ cells/ml compared to 7 × 10^6^cells/ml for CHO control host A. Similar trends were also observed for H_2_O_2_ evolved host B. In addition, viabilities remained significantly higher for H_2_O_2_ evolved hosts A and B compared to CHO control hosts A and B, although viabilities at Day 12 were comparably low between both hosts (Figure 7b,g). Second, all hosts displayed favorable lactate profiles with H_2_O_2_ evolved hosts A and B having lower lactate levels throughout the majority of the fed‐batch process (Figure 7c,h). Finally, the titers of BisAb A were 3.5‐fold higher from H_2_O_2_ evolved host A compared to the CHO control host (Figure 7d). Improvements in titer were also seen for BisAb B where titers from the H_2_O_2_ evolved host were 1.75‐fold higher than the CHO control host (Figure 7i). Interestingly, H_2_O_2_ evolved host A demonstrated a significant increase in specific productivity (qP) of 1.6‐fold compared to CHO control A (Figure 7e). Indeed, the increased volumetric titer observed for BisAb A is likely derived from a combination of improved qP as well as cell growth and viability. By contrast, the improved titer seen for BisAb B appears to result from improved growth and viability as qP was not significantly different between hosts (Figure 7j). In addition, intracellular expression of BisAb A and B, assessed by flow cytometry, showed comparable profiles for Hc and Lc in both CHO control and H_2_O_2_ evolved host cells (data not shown).

## 4 DISCUSSION

The production of DTE biopharmaceuticals by manufacturing cell lines is often hindered by low product yields (Spiess et al., 2015) that are, in some cases, associated with enhanced cellular stress. One such stress pertains to alterations in cellular redox state where elevated ROS generation arises due to a complex interplay between ER and mitochondrial burden (Templeton et al., 2013; Tu & Weissman, 2004; Turrens, 2003) as well as fluctuations in bioreactor conditions such as changes in dissolved oxygen concentrations (Handlogten et al., 2018, 2020) and cell culture medium components (Halliwell, 2014; Kelts et al., 2015; Schnellbaecher et al., 2019). The subsequent accumulation of ROS can damage the cell leading to poorer cell performance and lower antibody titers. To address these challenges, we generated a novel H_2_O_2_ evolved host that was evaluated for the expression of two industrially relevant DTE BisAbs. This host demonstrated heritable resistance to H_2_O_2_ (Figure 1a,b) and improved survival in the presence of several prooxidant chemicals that were selected to mimic bioreactor stressors (Figure 2a–d). These chemicals affect a diverse subset of intracellular redox pathways such as those involved in GSH biosynthesis and turnover (BSO and MS; Dunning et al., 2013; Lee et al., 1992), intracellular ROS generation (MSB; Beck et al., 2011), and hypoxia‐induced ROS production (CoCl; Zou et al., 2001). Consistent with this improved ability to resist oxidative stress, the H_2_O_2_ evolved cells also significantly upregulate several central antioxidant defense genes that combat ROS through multiple mechanisms (Figure 3c–f). These genes include the H_2_O_2_ scavenger, catalase, along with several enzymes associated with GSH production and activity (GCLM, GSS, and xCT). Indeed, this upregulation in genes involved in GSH synthesis correlated with a significant increase in the level of total GSH content in the H_2_O_2_ evolved host (Figure 3a). Interestingly, despite this increase in total GSH synthesis, both the H_2_O_2_ evolved host and CHO control cells were able to maintain redox homeostasis through a preserved GSH:GSSG ratio when in the non‐expressing state, this is potentially due to the absence of an oxidative stressor. By contrast, when the H_2_O_2_ evolved host was placed under stressful conditions such as those induced by the expression of DTE BisAbs, this resulted in an improved ratio of GSH:GSSG compared to CHO control cells (Figure 5a–d). Taken together, these data suggest that H_2_O_2_ directed evolution of our AZ proprietary CHO host has resulted in divergent expression of numerous antioxidant defense genes that renders the host capable of withstanding multiple sources of oxidative stress.

To investigate whether H_2_O_2_ directed evolution translated to improved cell performance, we evaluated the host at two stages of the cell line generation process associated with elevated cellular stress: recovery from stable transfection by electroporation and in a fed‐batch process. Following stable transfection with plasmid DNA encoding two distinct BisAbs, the H_2_O_2_ evolved host demonstrated faster recovery (11 days posttransfection) compared to CHO control host cells (14 days posttransfection) both in terms of viability and VCD (Figure 6a,b). When the cellular performance was assessed in a fed‐batch process, H_2_O_2_ evolved hosts A and B also displayed significantly improved viability and growth compared to CHO control hosts A and B. In addition, significantly higher BisAb titers were observed with the H_2_O_2_ evolved host, which for H_2_O_2_ evolved host A was due to a combined effect of improved growth and viability as well as significantly elevated qP (Figure 7).

The improved expression of DTE BisAbs in the H_2_O_2_ evolved host appears to be linked to enhanced antioxidant capacity. Indeed, oxidative stress induced by MSB was better tolerated by H_2_O_2_ evolved hosts A and B compared to CHO control hosts A and B (Figure 4a,b). Interestingly, previous studies have drawn links between the expression of antioxidants such as peroxiredoxin 5 and mnSOD and improved CHO cell survival in response to oxidative stress (Banmeyer et al., 2004; Warner et al., 1993). Perhaps consistent with these observations, H_2_O_2_ evolved hosts A and B also demonstrated a significant upregulation in a panel of diverse antioxidant defense genes such as those involved with GSH biosynthesis and turnover (GCLC, GCLM, GSS, and GPrx; Figure 5). Perhaps unsurprisingly, enhanced expression of GCLM and GSS have been linked to high producer cell lines (Orellana et al., 2015), and GCLM overexpression has been shown to improve mAb production (Orellana et al., 2017). Furthermore, we observed a significant upregulation in total GSH in H_2_O_2_ evolved hosts A and B (Figure 5a–d). This phenotype observed in the H_2_O_2_ evolved host is supported by data linking increased GSH content with improved mAb titers (Chong et al., 2012). In addition, Geoghegan et al demonstrated that the mRNA expression of the cysteine transporter, xCT, was upregulated in CHO cells during increased mAb production in the stationary phase of growth. Moreover, this phenotype was sensitive to xCT inhibition by sulfasalazine and linked to oxidative stress induced by high mAb production (Geoghegan et al., 2018). These data taken together with data in this study, suggest that elevated xCT expression in the H_2_O_2_ evolved host may be important for boosting BisAb titers and/or responding to oxidative stress.

Interestingly the ER‐resident protein, ER oxidoreductin 1 (ERO1), is known to oxidize protein disulfide isomerase leading to the formation of H_2_O_2_ during the protein folding process. An increased folding rate, such as that which occurs during recombinant protein expression can therefore trigger excessive H_2_O_2_ production within the ER (Zhang & Kaufman, 2008). Indeed, it is apparent from data presented here that the H_2_O_2_ evolved host displays significantly elevated catalase expression in both non‐expressing and expressing states (Figures 3e and 5g,i). Although catalase activity was not measured here, previous studies have demonstrated that catalase activity could be enhanced using directed host cell evolution (Spitz et al., 1988) and may facilitate the elimination of excessive H_2_O_2_ production, therefore preventing oxidative stress. In addition to the findings presented here, a recent transcriptomic analysis performed by our group in which non‐expressing H_2_O_2_ evolved host cells were compared with CHO control cells (data not shown) revealed that in addition to the upregulation of diverse antioxidant genes there was a concomitant downregulation of a number of pro‐oxidant genes. These data further exemplify the importance of modulating diverse cellular pathways, as opposed to the targeted expression of single genes, when engineering cells to improve the manufacture of biotherapeutics. It is also important to highlight that in addition to improved antioxidant capacity other mechanisms, not investigated here, may also be involved in improving BisAb titers such as elevated transcription of antibody genes or increased gene copy number. Collectively, our data suggest that the H_2_O_2_ evolved host is better equipped to combat excessive H_2_O_2_ production and that H_2_O_2_‐induced evolution can improve cell performance through alterations in the expression of diverse redox pathways. Indeed, given that these phenotypes appear to be stable in transfection pools, which for CHO cells are known to exhibit considerable phenotypic instability, we anticipate that these beneficial phenotypes will also be maintained upon the generation of more phenotypically stable clonal cell populations, although this remains to be investigated.

To conclude, we have generated a novel H_2_O_2_ evolved CHO host that has been evaluated for the expression of two industrially relevant DTE BisAbs. The data presented here indicate that global changes in antioxidant pathways, such as those involved in GSH biosynthesis and turnover as well as H_2_O_2_ elimination, can confer cellular resistance to a diverse subset of oxidative stressors. Moreover, boosting antioxidant capacity appears to have advantages for better cell growth, viability, and DTE BisAb titers. Improving the expression of DTE biotherapeutics is of great importance as the range of novel formats in the biopharmaceutical industry is expanding rapidly and poses great challenges for their developability and manufacture. By using a host that is better equipped to deal with these stresses alleviates one such challenge. This study highlights the beneficial effects of directed host cell evolution in augmenting global cellular redox networks to improve the manufacturing of DTE biotherapeutics. Finally, these data offer insights into the role of cellular antioxidants in the production cell lines and therefore support growing research efforts to control cellular redox and boost recombinant protein production.

## CONFLICT OF INTERESTS

The authors declare that there are no conflict of interests.

## AUTHOR CONTRIBUTIONS

Rajesh K. Mistry contributed mechanistic data and wrote the manuscript with the support of Emma Kelsall, Si N. (Susie) Sou, Diane Hatton, and Suzanne Gibson. Emma Kelsall and Harriet Barker contributed fed‐batch data; Si N. (Susie) Sou contributed stability data; Katie Willis and Mike Jenns contributed to host generation; Fabio Zurlo contributed titer analysis.
